# The prognostic role of PD-1, PD-L1, ALK, and ROS1 proteins expression in non-small cell lung carcinoma patients from Egypt

**DOI:** 10.1186/s43046-022-00121-8

**Published:** 2022-05-30

**Authors:** Abeer A. Bahnassy, Hoda Ismail, Marwa Mohanad, Ahmed El-Bastawisy, Hend F. Yousef

**Affiliations:** 1 Tissue Culture and Cytogenetics Unit, Pathology Department, National Cancer Institute, Cairo University, Cairo, 11976 Egypt; 2 Surgical Pathology Department, National Cancer Institute, Cairo University, Cairo, 11976 Egypt; 3 Biochemistry Department, College of Pharmaceutical Sciences and Drug Manufacturing, Misr University for Science and Technology, 6th of October, 12945 Egypt; 4 Medical Oncology Department, National Cancer Institute, Cairo University, Cairo, 11976 Egypt

**Keywords:** Non-small cell lung carcinoma, Programmed death-1/programmed-death-ligand-1, Anaplastic lymphoma kinase (ALK), c-ros oncogene1 (ROS1), Prognosis

## Abstract

**Background:**

Programmed death ligand-1 (PD-L1), anaplastic lymphoma kinase (ALK), and c-ros oncogene1 (ROS1) expression may influence the prognosis of non-small cell lung carcinoma (NSCLC). We aimed to investigate the prognostic and predictive significance of PD-1/PD-L1 along with c-ros ROS1 and ALK in NSCLC patients.

**Methods:**

Immunohistochemistry used to identify ALK, ROS1, PD-1, and PD-L1 proteins expression as well as *ROS1* rearrangement via fluorescence in situ hybridization, in 70 NSCLC patients. Results were related to clinicopathological feature, survival, and treatment response.

**Results:**

Expression of ROS1, ALK, PD-1, and PD-L1 and ROS1-rearrangement were detected in 18.57%, 54.29%, 84.29%, 87.14%, and 15.71% of the cases, respectively. No association was found between ROS1, PD-1, and PD-L1 and any clinicopathological features, survival, or treatment outcome. ALK expression significantly associated with stage-IV and left-sided tumors. Epidermal growth factor receptor *(EGFR)* mutation and ALK-positive patients had significantly reduced progression-free survival than patients with wild type EGFR [HR: 1.99, 95% CI: 1.37–2.93, *p* < 0.001] and negative-ALK expression [HR: 1.46, 95% CI: 1.03–2.07, *p* = 0.03]. In multivariate analysis, lymph node metastasis, *EGFR*-mutations, and ALK were independent predictors of NSCLC. PD-L1 expression was significantly correlated with PD-1 but not with ROS1, ALK, or *EGFR-*mutation.

**Conclusion:**

Positive ALK expression and *EGFR*-mutations are independent adverse predictors of NSCLC. Overexpression of PD-1/PD-L1 is not a significant prognostic marker in NSCLC patients receiving chemotherapy, making them susceptible to immunotherapy. Since PD-1/PD-L1 expression is independent to oncogenic driver mutations, future studies into specific immune checkpoint inhibitors combined with targeted therapies for individualized treatment of NSCLC is warranted.

Positive ALK expression and EGFR mutations are independent risk factors for NSCLC. Overexpression of PD-1/PD-L1 is not a significant prognostic factor in patients with NSCLC who are receiving chemotherapy, making them immunotherapy susceptible. Given that PD-1/PD-L1 expression is not dependent on oncogenic driver mutations, additional research into specific immune checkpoint inhibitors in combination with targeted therapies for the treatment of NSCLC on an individual basis is warranted.

## Background

Non-small cell lung carcinoma (NSCLC) accounts for nearly 84% of all lung cancer cases. Despite rapid declines in lung cancer mortality rates, it remains the leading cause of cancer-related death worldwide [1]. Platinum-based chemotherapy is the first line treatment for NSCLC patients. However, regular platinum-based treatment has been linked to poor response rates and prognosis in NSCLC. There are also differences in how patients respond to chemotherapy based on their clinical characteristics, tumor stage, and survival rates [2]. This highlights the urgent need for novel molecular targets to predict NSCLC patients who may benefit from personalized therapy. The implementation of tailored NSCLC management based on molecular targets has improved patient outcomes [3].

Gene mutation, rearrangement, and gene amplification screening has emerged as a new avenue for extremely successful treatment approaches. NSCLC patients should have routine testing for EGFR, ALK, and ROS1 mutations and rearrangements, as well as KRAS viral oncogene mutations and rearrangements, as part of their molecular evaluation [4, 5]. Previous studies have indicated that ROS1 and ALK chromosomal rearrangements occur in just 1–2% and 3–4% of NSCLC, respectively. Both *ROS1* and *ALK* are transmembrane tyrosine kinase receptors with significant amino acid sequence homology in their respective tyrosine kinase domains [6].

Both *EGFR* and *ALK* targeted therapies were approved for treatment of metastatic NSCLC resulting in improved survival for respective patients with *EGFR* mutations and *ALK*-positive disease. Moreover, *ALK/ROS1/MET* inhibitors had improved the response rate and progression-free survival of *ALK* and *ROS1* positive advanced NSCLC patients. Despite the success of targeted therapies, the inevitable acquired resistance and the lack of oncogenic driver mutations in the majority of the patients pose a significant clinical challenge [7]. In recent years, immunotherapy utilizing a combination of programmed death protein-1/ programmed death ligand1 (PD-1/ PD-L1) blockade has emerged as one of the most promising anticancer immunotherapies for treatment of several types of cancer including the NSCLC. Unlike targeted therapy, immune checkpoint inhibitors now elicit objective responses in cancer patients regardless of their mutation status. However, these immunotherapies only treat a small subset of patients, with most developing acquired or intrinsic resistance [8]. Moreover, immune checkpoint inhibitors have been linked to a low response rate in NSCLC patients with *EGFR* or *ALK* aberrations. Thus, several clinical trials are currently assessing the combination of PD-1/PD-L1 inhibitors with EGFR and ALK tyrosine kinase inhibitors in advanced NSCLC [9]. Researchers discovered that *ROS1* gene rearrangement in NSCLC did not coincide with other oncogenic driver mutations including *EGFR, KRAS*, and *ALK*. However, other studies indicated that ROS1-positive NSCLC exhibited oncogenic driver mutations [10]. Thus, identifying PD-1/PD-L1 expression as well as driver gene mutations is critical for guiding personalized NSCLC treatment. To date, the correlation and concomitancy of PD1/PD-L1 expression with oncogenic driver alterations in Egyptian NSCLC patients has not been thoroughly investigated.

In the current study, we used immunohistochemistry (IHC) to investigate the of ALK, ROS1, PD-1, and PD-L1 proteins expression as well as detection of *ROS1* gene rearrangement by fluorescence in situ hybridization (FISH) technique in relation to relevant clinicopathological features of patients, survival rates, and response to treatment in a cohort of Egyptian NSCLC patients. This is critical for identifying accurate prognostic and predictive biomarkers that can accurately predict who will benefit from personalized therapies in patients with NSCLC.

## Methods

### Ethical approve committee

This retrospective study was approved by the Ethics Committee/Institutional Review Board (IRB) of the cancer institute, in accordance with the principles of the Declaration of Helsinki and Good Clinical Practice guidelines. Written informed consent forms were obtained from each patient for sampling and research.

### Patients and tumor specimens

Tissue samples were recruited from 70 NSCLC patients who were diagnosed and treated in the period from May 2015 to November 2016. All tumor samples were routinely fixed in 10% neutral buffered formalin (pH 7.4) for 24–48 h before being embedded in paraffin. An experienced pathologist used thyreoid-transcription-factor-1 (TTF-1) and p40 diagnosed for precise pathohistological diagnosis of NSCLC histological subtypes.

TTF-1 is a lung adenocarcinoma diagnostic marker, whereas p40 is a squamous cell carcinoma diagnostic marker [11].

The clinicopathological features of patients including age, gender, tumor size, stage, grade, lymph node (LN) metastasis, and EGFR mutations were collected from the medical hospital and pathology department (Table 1). Bronchoscopic examinations were performed on a routine basis. The data were analyzed anonymously where patients’ private information was not released. Tumors were pathologically staged using the 8^th^ edition of the lung cancer tumor–node–metastasis (TNM) classification [12]. Lymph nodes measuring longer than 1 cm in the shortest axis diameter on CT scanning were considered positive LN metastasis preoperatively. Post-operative pathological assessment of LN metastasis was performed using hematoxylin and eosin staining. N0: no lymph node metastasis. N1: any positive metastatic lymph nodes in station 10–14. N2: any positive metastatic lymph nodes in station 2–9. Patients with a second malignancy, those who had previously received chemotherapy, or those who were currently enrolled in another clinical trial were excluded from the current study.

**Table 1:** Patient’s characteristics

Characteristics	
Age (years), mean ± SD	55.2 ± 9.8
**Age,** ***N*** **(%)**	
< 55	32 (45.7)
≥ 55	38 (54.3)
**Gender,** ***N*** **(%)**	
Female	19 (27.1)
Male	51 (72.9)
**Smoking status,** ***N*** **(%)**	
Never-smoker	27 (38.6)
Current/ex-smoker	43 (61.4)
**Grade,** ***N*** **(%)**	
1–2	51 (72.9)
3	19 (27.1)
**Stage,** ***N*** **(%)**	
I	12 (17.1)
II	15 (21.4)
III	12 (17.1)
IV	31 (44.3)
**Tumor size (cm), mean ± SD**	5.4 ± 2.78
**Tumor size (cm),** ***N*** **(%)**	
< 4	25 (35.7)
≥ 4	45 (64.3)
**Laterality,** ***N*** **(%)**	
Left	36 (51.4)
Right	34 (48.6)
**LN metastasis,** ***N*** **(%)**	
No	34 (48.6)
Yes	36 (51.4)
**EGFR mutation,** ***N*** **(%)**	
Wild type	40 (57.1)
Mutant	21 (30.0)
Unknown	9 (12.9)

*LN* lymph node, *EGFR* epidermal growth factor receptor

### Treatment protocol

Fifty-seven patients (81.4%) underwent surgical resection of tumors. Chemotherapy was given as neoadjuvant treatment to 25 patients (35.7%). Adjuvant chemotherapy was given to 34 patients (48.57%). Patients with a locally advanced stage received two to four cycles of platinum-based adjuvant chemotherapy (platinum–pemetrexed, taxol, or docetaxel). The chemotherapy treatment regimen was 1000 mg/m^2^ IV gemcitabine on 250 cc normal saline (NS) over 30 min for 8 days and 80 mg/m^2^ IV cisplatin on 500 cc NS over 1 h for 1 day every 3 weeks up to 6 cycles for the responding patients.

### Follow-up and treatment assessment

The initial pretreatment evaluation included a thorough medical history as well as a physical examination. Furthermore, vital signs, a complete blood count (CBC) with a differential and full biochemical panel, liver, and renal function, were repetitively evaluated prior each treatment course.

The efficacy of response to treatment was evaluated according to the updated Response Evaluation Criteria in Solid Tumors (RECIST) version 1.1 [13]. A complete response (CR) is defined as the absence of all known disease as determined by two observations no less than four weeks apart. A partial response (PR) is defined as a reduction of 30% or more in the product of the perpendicular diameters of all measurable lesions. Stable disease (SD) denotes a decrease by less than 30% or increase by less than 20% in tumor size. Progressive disease (PD) was defined as an increase of more than 20% in the product of all measurable lesions’ perpendicular diameters or the appearance of new lesions.

In case of intolerable or worse adverse effects, the treatment was modified or substantially discontinued. Radiologic evaluations using computed tomography (CT) scans were performed at the beginning of the intervention and then once every 8 weeks until disease progression. The assessment was repeated 4–8 weeks after the initial response to confirm the response rates. All patients were followed up on every 12 weeks for treatment responses and survival until death or study completion. Every 4 weeks, patients’ safety was evaluated. The Common Terminology Criteria for Adverse Events (CTCAE), version 6.0 [14], was used to grade AEs, laboratory tests, and vital signs.

The follow-up deadline was November 2020. By the end of follow-up, progression-free survival (PFS) was measured in months from the date of primary surgery or treatment to the first time of progressive disease and censored at the date of last follow-up for survivors without progression.

### Immunohistochemical analysis

Archival paraffin blocks were obtained from the Pathology Department for each of the 70 cases assessed. Hematoxylin and eosin (H&E) slides were used to identify the most representative paraffin blocks, and only the cells with more than 75% neoplastic cells were included in the study. From each tumor block, 4 sections (5 μm each) were mounted onto positive-charged slides for ALK, ROS1, PD-1, and PD-L1 (Fisher), and another section (5 μm) was used for detection of *ROS1* rearrangement by FISH.

Briefly, the sections were deparaffinized in xylene and hydrated in a series of ethanol gradient. Antigen retrieval was carried out in a microwave at 98 °C for 30 min in a citrate buffer pH 6.0. Endogenous peroxidase activity was hindered by treatment with 3.0% H_2_O_2_, which was followed by blocking of non-specific antibody binding using 1.5% blocking serum (Santa Cruz Biotechnology, Santa Cruz, CA, USA) for 2 h at room temperature in phosphate-buffered saline (PBS). Subsequently, the slides were incubated overnight with anti-ROS1 (4-6G, ab108492, abcam, 1:100), anti-ALK (C-terminal, ab190934, abcam), anti*-*PD-1 (CAL15, ab237727, abcam, 1:1000), and anti-PD-L1 (BLR020E, ab243877, abcam, dilution 1:100) according to manufacturer’s protocol. Sections were then washed three times in PBS, incubated with Envision reagent (Dako), followed by color development with diaminobenzidine (DAB) reagent (abcam). Finally, the slides were counterstained with hematoxylin, dehydrated with ethanol, cleaned with xylene, and examined microscopically.

Five high-power fields (40x) were randomly chosen for each sample. ROS1 and PD-1 protein scoring were graded as follows: 0, no expression or nuclear expression only; 1+, cytoplasmic was faint; 2+, cytoplasmic staining was present in 0 to 50% of tumor cells; and 3+, cytoplasmic staining exceeding in > 50% of tumor cells [15]. Positive cases demonstrated a granular to diffuse cytoplasmic expression pattern, frequently of varying intensity within the tumor cell population. ALK protein expression was scored according to membranous or cytoplasmic staining as follows: 0, negative or no staining; 1+, faint; 2+, moderate; and 3+, strong staining intensity in at least 10% tumor cells [16]. PDL-1 protein expression was determined by using Tumor Proportion Score (TPS) which is the percentage of viable tissue cells showing partial or complete membrane staining at any intensity [17]. Three balanced groups were used to score the PD-L1 staining: negative TPS (1% or absence of reactivity), intermediate expressors (1–49% of tumor cells), and strong expressors (50% of tumor cells). For statistical purposes, a case was considered positive if at least 50% of tumor cells had brown membranous and/or cytoplasmic monoclonal antibody immunostaining for ROS1, PD-1, and PD-L1 and at least 10% of tumor cells had ALK immunostaining.

### ROS1 rearrangement by fluorescence in situ hybridization (FISH)

Fluorescence in situ hybridization (FISH) was performed on FFPE using 6q22 *ROS1* Break Apart FISH Probe RUO Kit (Abbott Molecular Inc., IL, USA) according to manufacturer’s instructions. Four microliters were deparaffinized in xylene followed by hydration in a series of ethanol gradient. Slides were then heated in boiled water for 30 min before being digested with proteinase K (37 °C, 5 min). This was followed by washing in 2X saline sodium citrate (SSC) and dehydration in an increasing ethanol gradient (70%, 85% and 100%) for 3–5 min each. After air drying, the target specimens and the FISH probe were incubated in humidified hybridization machine and the hybridization was carried out as follows, denaturation at 75 °C for 8 min, followed by hybridization at 42 °C for 16 h. The slides were then washed in a 2X SSC and NP40 solution at 42 °C for 5 min before being immersed in 70% ethanol for 5 min. Then, 15 μL DAPI was used to counterstain. The fluorescence signals were examined in the dark using an Olympus BX53 fluorescence microscope (Olympus, Tokyo, Japan). The FISH positive for *ROS1* gene was defined as more than 15% tumor cells showing split signals (“orange” and “green” split signals) or isolated 3′ “green” signals belonged to the *ROS1* fusion rearrangement. On the other hand, *ROS1* rearrangement negative were defined as cells with intact fusion signal or with isolated 5′ “orange” signals.

### Statistical analysis

All statistical analyses were performed using Statistical Package for the Social Sciences (SPSS) version 22.0 (SPSS, Inc., Chicago, IL, USA) and Graph pad prism version 8.0. Sample size was calculated using G* Power data analysis software (IDRE stating, UCLA) adjusted for appropriate power of 0.8 and *α* error of 0.05. Fisher exact and chi-square tests were used to assess the relationship between ROS1, ALK, PD-1, and PD-L1 protein expression as well as *ROS1* rearrangement with clinicopathological features and response to treatment. Spearman rho was used to determine the correlation between ROS1, ALK, PD-1, and PD-L1 protein expression with each other and with *ROS1* rearrangement. The PFS analysis in association with protein expression of studied proteins was detected using Kaplan-Meier curve and log rank test. Cox regression was used univariate and multivariate survival analysis. *P* values less than 0.05 were considered statistically significant.

## Results

### Patients’ characteristics

Table 1 shows the clinicopathological characteristics of NSCLC patients whose specimens were submitted for analysis of *R*OS1, ALK, PD-1, and PD-L1 protein expressions, along with *ROS1* gene rearrangement. The mean age of patients was 55.2 ± 9.8 (range, 30–74) years, with 51 (72.9%) males and 19 (27.1%) females. The ratio of current/ex-smokers to never smokers was 43:27. The average tumor size was 5.4 ± 2.7 cm, with 36 (51.4%) of the cases have left-sided tumors and 34 (48.6%) having a right-sided tumors. Most of the cases (43/70, 61.43%) were advanced stage, while 27/70 (38.57%) were early stage (I–II). Fifty-one cases were grade1, 2 were grade 2 (72.9%), and 19 (27.1%) were grade 3. Thirty-six cases (51.4%) had LN metastasis, and 21 (30.0%) cases had *EGFR* mutations.

### Expression of ROS1, ALK, PD-1, and PD-L1 proteins

**Fig. 1:**
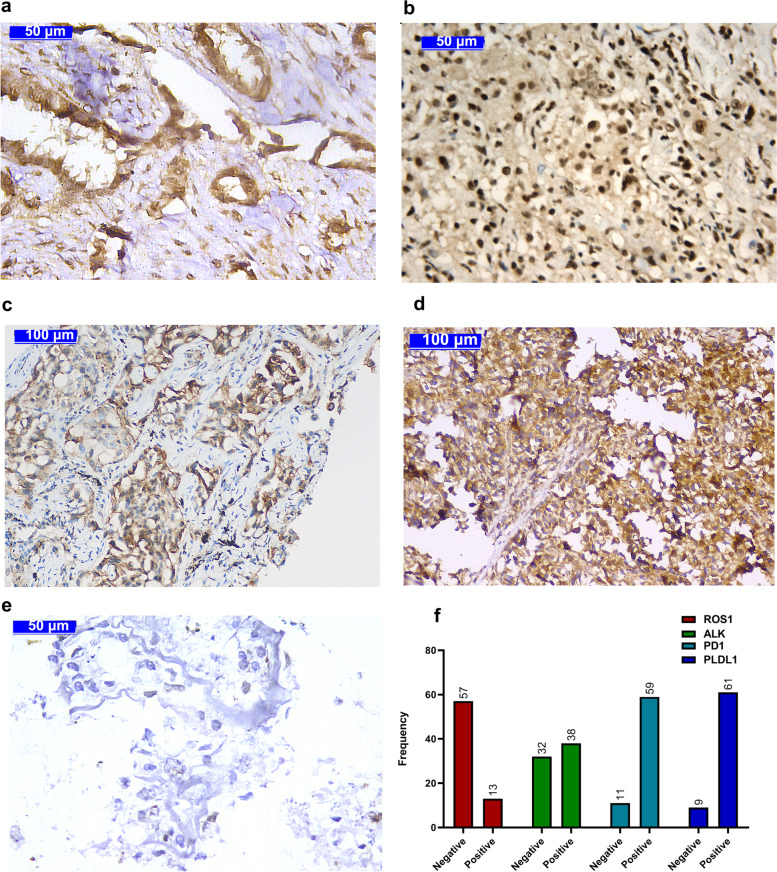
Immunohistochemical staining of **a** ROS1 (40X), **b** ALK (40X), **c** PD-1 (20X), and **d** PD-L1 (20X) in adenocarcinoma specimens. **e** Negative immunohistochemical expression (40X). Majority of cells showed membranous staining for ROS1, nuclear and membranous staining for ALK, cytoplasmic and membranous staining for PD-1 was and PD-L1. **f** Bar plot showing the frequency of the ROS1, ALK, PD-1, and PD-L1 proteins expression

Immunohistochemistry was used to assess the expression levels of ROS1, ALK, PD-1, and PD-L1 in the nucleus, cytoplasm, and/or the cell membrane of the specimens obtained from 70 NSCLC cases (Fig. 1). Protein expression was detected by IHC analysis. ROS1 protein expression was present in 13 (18.57%) cases, ALK protein expression in 38 (54.29%) cases, PD-1 protein in 59 (84.29%) cases, and PD-L1 protein expression in 61 (87.14%) of the cases.

### Clinicopathological characteristics in association with ROS1, ALK, PD-1, and PD-L1 proteins expressions

Positive ALK expression was significantly higher among patients with left-sided tumors (63.2%) compared to those with right-sided (36.8%) tumors (*p* = 0.03). Stage IV associated significantly with positive expression of ALK (24/38, 63.2%) as compared to stage I (2/38, 5.3%), stage II (6/38 15.8%), and stage III (6/38, 15.5%) (*p* = 0.002). No significant association was detected between ROS1, PD-1, or PD-L1 protein expressions and any of the clinicopathological characteristics of the cases assessed (Table 2).

**Table 2:** Clinicopathological characteristics in association with ROS1, ALK, PD-1, and PD-L1 proteins expression

		ROS1		ALK		PD-1		PD-L1	
	No	-ve	+ve	-ve	+ve	-ve	+ve	-ve	+ve
**Age**									
< 55	32	27 (84.4)	5 (15.6)	14 (43.75)	18 (56.25)	4 (12.5)	28 (87.5)	4 (12.5)	28 (87.5)
≥ 55	38	30 (78.9)	8 (21.1)	18 (47.4)	20 (52.6)	7 (18.4)	31 (81.6)	5 (13.2)	33 (86.8)
^*a*^*p*-value		0.76		0.81		0.53		1.00	
**Gender**									
Female	19	16 (84.2)	3 (15.8)	9 (47.4)	10 (52.6)	5 (26.3)	14 (73.7)	4 (21.1)	15 (78.9)
Male	51	41 (80.4)	10 (19.6)	23 (45.1)	28 (54.9)	6 (11.8)	45 (88.2)	5 (9.8)	46 (90.2)
^*a*^*p*-value		1.00		1.00		0.15		0.24	
**Smoking status**									
Never-smoker	27	22 (81.5)	5 (18.5)	13 (48.1)	14 (51.9)	5 (18.5)	22 (81.5)	3 (11.1)	24 (88.9)
Current/ex-smoker	41	35 (81.4)	8 (18.6)	19 (44.2)	24 (55.8)	6 (13.95)	37 (86.05)	6 (13.95)	37 (86.05)
^*a*^*p*-value		1.00		0.81		0.74		1.00	
**Grade**									
1–2	51	41 (80.4)	10 (19.6)	24 (47.1)	27 (52.9)	7 (13.7)	44 (86.3)	8 (15.7)	43 (84.3)
3	19	16 (84.2)	3 (15.8)	8 (42.1)	11 (57.9)	4 (21.1)	15 (78.9)	1 (5.3)	18 (94.7)
^*a*^*p*-value		1.00		0.79		0.47		0.43	
**Stage**									
I	12	8 (66.7)	4 (33.3)	10 (83.3)	2 (16.7)	3 (25.0)	9 (75.0)	1 (8.3)	11 (91.7)
II	15	13 (86.7)	2 (13.3)	9 (60.0)	6 (40.0)	3 (20.0)	12 (80.0)	3 (20.0)	12 (80.0)
III	12	11 (91.7)	1 (8.3)	6 (50.0)	6 (50.0)	2 (16.7)	10 (83.3)	1 (8.3)	11 (91.7)
IV	31	25 (80.6)	6 (19.4)	7 (22.6)	24 (77.4)	3 (9.7)	28 (90.3)	4 (12.9)	27 (87.1)
^*b*^*p*-value		0.42		0.002**		0.60		0.77	
**Tumor size (cm**									
< 4	25	21 (84.0)	4 (16.0)	14 (56.0)	11 (44.0)	2 (8.0)	23 (92.0)	4 (16.0)	21 (84.0)
≥ 4	45	36 (80.0)	9 (20.0)	18 (40.0)	27 (60.0)	9 (20.0)	36 (80.0)	5 (11.1)	40 (88.9)
^*a*^*p*-value		0.76		0.22		0.31		71.2	
**Laterality**									
Left	36	27 (75.0)	9 (25.0)	12 (33.3)	24 (66.7)	3 (8.3)	33 (91.7)	2 (5.6)	34 (94.4)
Right	34	30 (88.2)	4 (11.8)	20 (58.8)	14 (41.2)	8 (23.5)	26 (76.5)	7 (20.6)	27 (79.4)
^*a*^*p*-value		0.22		0.04*		0.10		0.08	
**LN metastasis**									
No	34	28 (82.4)	6 (17.6)	17 (50.0)	17 (50.0)	5 (14.7)	29 (85.3)	5 (14.7)	29 (85.3)
Yes	36	29 (80.6)	7 (19.4)	15 (41.7)	21 (58.3)	6 (16.7)	30 (83.3)	4 (11.1)	32 (88.9)
^*a*^*p*-value		1.0		0.63		1.0		0.73	
***EGFR*** **mutation**									
Wild type	40	32 (80.0)	8 (20.0)	19 (47.5)	21 (52.5)	8 (20.0)	32 (80.0)	6 (15.0)	34 (85.0)
Mutant	21	17 (80.95)	4 (19.05)	7 (33.3)	14 (66.7)	2 (9.5)	19 (90.5)	2 (9.5)	19 (90.5)
Unknown	9	8 (88.9)	1 (11.1)	6 (66.7)	3 (33.3)	1 (11.1)	8 (88.9)	1 (11.1)	8 (88.9)
^*b*^*p*-value		0.82		0.23		0.52		0.82	

*LN* lymph node, *EGFR* epidermal growth factor receptor, *ROS1* c-ros oncogene1, *ALK* anaplastic lymphoma kinase, *PD-1* programmed cell death-1, *PD-L1* programmed cell death ligand-1

**Significant at *p* < 0.01

^a^Fisher exact test was used

^b^Chi-square test was used

### ROS1 rearrangement as detected by FISH

**Fig. 2:**
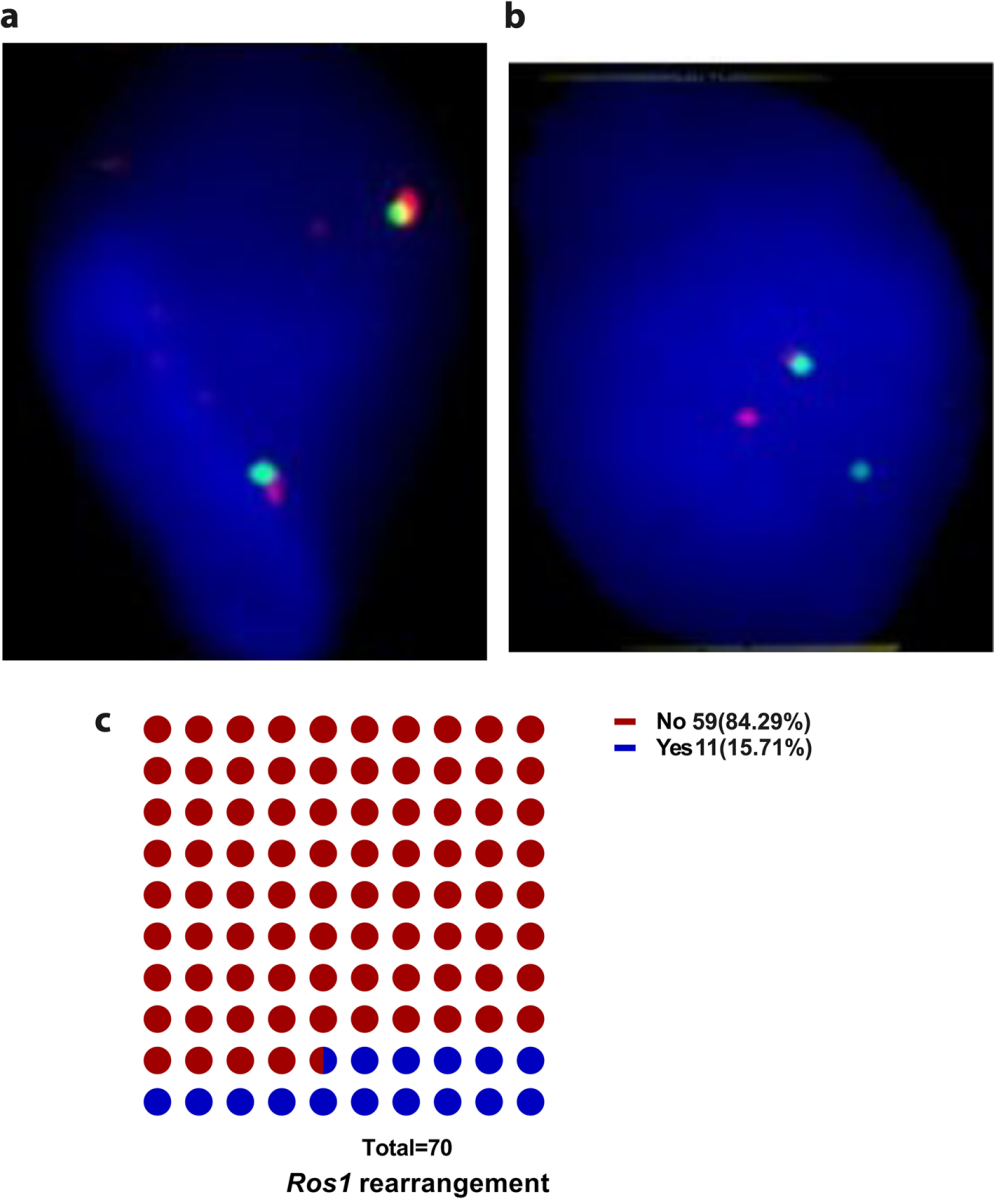
Detection of *ROS1* gene rearrangement using FISH. **a** FISH-negative case showing intact two fused signals per nucleus. **b** FISH-positive cases representing split (red and green) signals. Original magnification × 1000. **c** Bar plot showing the frequency of the *ROS1* gene rearrangement

*ROS1* rearrangements were identified in 11 out of the NSCLC cases with overall positive rate of (11/70, 15.71%) (Fig. 2). No significant association was found between *ROS1* rearrangement and any of the clinic-pathological features of the patients (Table 3).

**Table 3:** Clinicopathological characteristics in association with *ROS1* gene rearrangement

		***ROS1*** FISH		***p-***value
	*N*	-ve	+ve	
**Age**				
< 55	32	26 (81.25)	6 (18.75)	0.74^a^
≥ 55	38	33 (86.80)	5 (13.20)	
**Gender**				
Female	19	17 (89.50)	2 (10.50)	0.71^a^
Male	51	42 (83.35)	9 (16.65)	
**Smoking status**				
Never-smoker	27	23 (85.20)	4 (14.80)	1.00^a^
Current/ex-smoker	43	36 (83.70)	7 (16.30)	
**Grade**				
1–2	51	43 (84.30)	8 (15.70)	1.00^a^
3	19	16 (84.20)	3 (15.80)	
**Stage**				
I	12	8 (66.67)	4 (33.33)	0.24^b^
II	15	14 (93.30)	1 (6.70)	
III	12	11 (91.67)	1 (8.33)	
IV	31	26 (83.80)	5 (16.20)	
**Tumor size (cm)**				
< 4	25	21 (84.00)	4 (16.00)	1.00^a^
≥ 4	45	38 (84.40)	7 (15.60)	
**Laterality**				
Left	36	28 (77.80)	8 (22.20)	0.19^a^
Right	34	31 (91.20)	3 (8.80)	
**LN metastasis**				
No	34	29 (85.30)	5 (14.70)	1.00^a^
Yes	36	30 (83.33)	6 (16.67)	
**EGFR mutation**				
Wild type	40	35 (87.50)	5 (12.50)	0.47^b^
Mutant	21	16 (76.20)	5 (23.80)	
Unknown	9	8 (88.90)	1 (11.10)	

*LN* lymph node, *EGFR* epidermal growth factor receptor, *ROS1* c-ros oncogene1

^a^Fisher exact test was used

^b^Chi-square test was used

### Survival analysis in association with ROS1, ALK, PD-1, and PD-L1 proteins expressions and ROS1 rearrangement

**Fig. 3:**
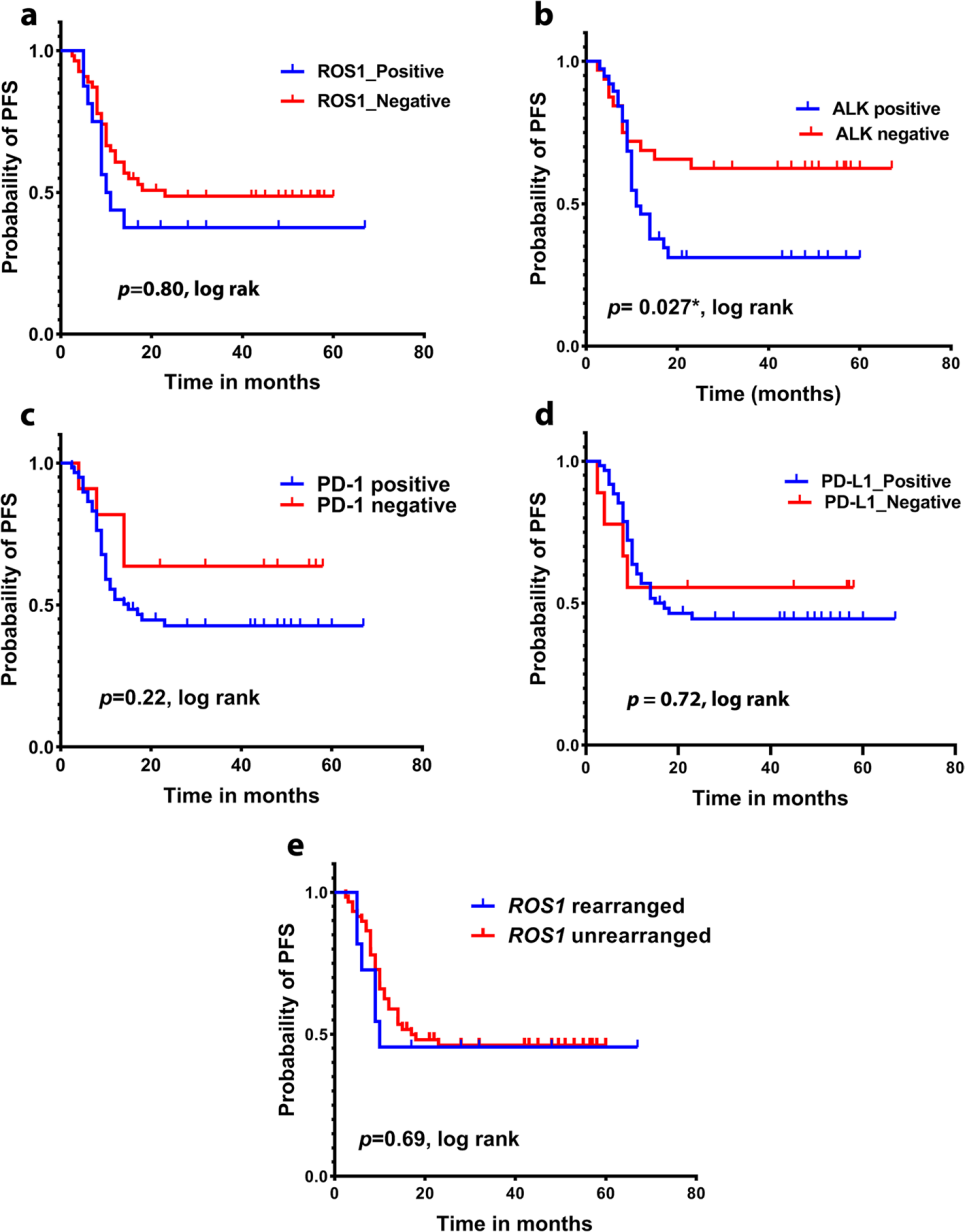
Kaplan-Meier survival analysis curve for the progression free survival (PFS) in relation to expression of **a** ROS1, **b** ALK, **c** PD-1, and **d** PD-L1 and in relation to **e** *ROS1* rearrangement

The median follow-up survival was 20.5 months (range, 10–67). Positive expression of ALK protein was significantly associated with reduced PFS in NSCLC patients. Kaplan-Meier survival analysis (Fig. 3) revealed that the median PFS for patients with negative ALK expression was undefined (more than 50% of the cases were censored at the time of last follow-up), whereas the median PFS for patients with positive ALK expression was 11 months, with a hazard ratio (HR) of 2.09 (95% CI: 1.10–4.14) (*p* = 0.027). There was no significant correlation between ROS1, PD-1, or PD-L1 protein expressions and the PFS in NSCLC patients. The median PFS of patients with negative ROS1, PD-1, and PD-L1 expression was 14 months, undefined, and 23 months, respectively compared to 17 months, 15 months, and 17 months, respectively, for patients with positive expression of respective proteins (*p* = 0.80, 0.22 and 0.72, log rank, respectively), with a HR of 0.90 (95% CI: 0.36–2.22), 1.85(95% CI: 0.81–4.26), and 1.19 (95% CI: 0.47–3.01), respectively.

There was no significant correlation between *ROS1* rearrangement and the survival outcome in patients with NSCLC. The median PFS for patients with *ROS1* rearrangement was 10 months, whereas the median PFS for patients without *ROS1* rearrangement was 17 months (*p* = 0.69, log rank), with a HR of 1.19 (95% CI: 0.47–3.01).

### Univariate and multivariate survival analysis

Univariate Cox regression survival analysis revealed that positive LN metastasis, *EGFR* mutations, and positive ALK protein expression were significantly associated with decreased PFS in NSCLC patients. Multivariate survival analysis revealed that LN metastasis, ALK protein expression, and *EGFR* mutations were independent predictors of PFS in patients with NSCLC (Table 4).

**Table 4:** Univariate and multivariate survival analysis for NSCLC patients

	PFS		
	HR	95% CI	*p*-value
**Univariate**			
Age	1.13	0.59–2.18	0.71
Gender	0.84	0.40–1.73	0.63
Smoking	0.73	0.38–1.40	0.35
Grade	1.04	0.51–2.10	0.92
T.size	1.62	0.78–3.35	0.19
Laterality	1.29	0.93–1.79	0.13
LN metastasis	1.65	1.17–2.34	0.005**
*EGFR* mutation	1.99	1.37–2.93	< 0.001***
ROS1	0.95	0.61–1.47	0.81
ALK	1.46	1.03–2.07	0.03*
PD-1	1.36	0.81–2.29	0.24
PD-L1	1.20	0.84–1.73	0.31
*ROS1* rearrangement	1.09	0.70–1.69	0.70
**Multivariate**			
Laterality			
LN metastasis	1.59	1.11–2.25	0.010*
ALK	1.46	1.03–2.08	0.04*
*EGFR*	2.08	1.37–3.15	< 0.001***

*HR* hazard ratio, *PFS* progression-free survival, *LN* lymph node, *EGFR* epidermal growth factor receptor, *ROS1* c-ros oncogene1, *ALK* anaplastic lymphoma kinase, *PD-1* programmed cell death-1, *PD-L1* programmed cell death ligand-1

Cox regression analysis was used

***Significant at *p* < 0.001. **Significant at *p* < 0.01. *Significant at *p* < 0.05

### Response to treatment

**Fig. 4:**
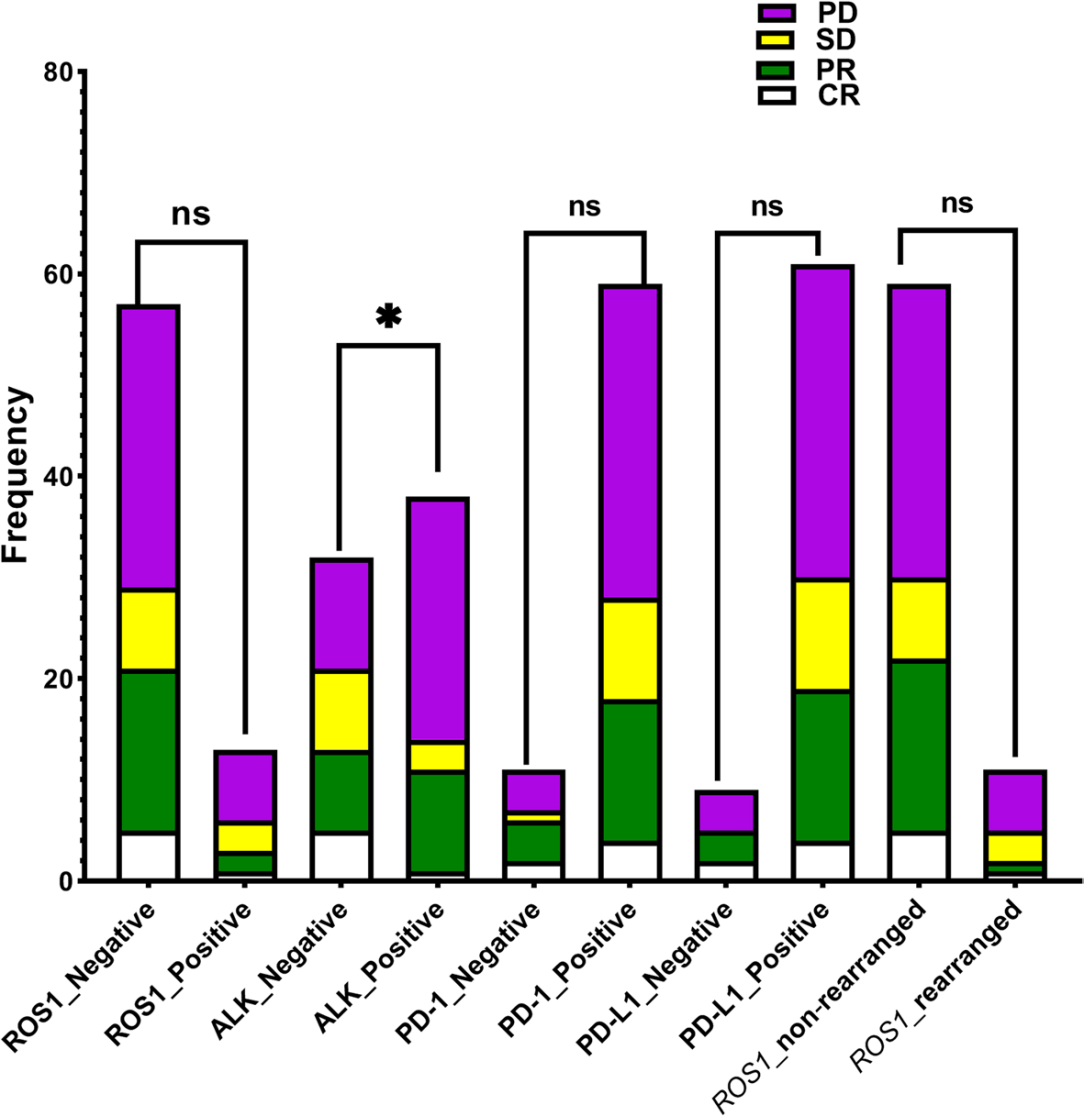
Bar plot showing the response rate in relation to ROS1, ALK, PD-1, and PD-L1 protein expression as well as to *ROS1* gene rearrangement. *Significant at *p* < 0.05

Six (8.57%) patients with NSCLC had CR, 18 (25.71%) had partial response, 11 (15.71%) had SD, and 35 (50.0%) had a PD. There was no significant relationship between ROS1, PD-1, or PD-L1 protein expression, as well as between *ROS1* gene rearrangement and treatment response. However, positive ALK expression was significantly frequent in patients with PD (24/35. 68.6%) compared to those with CR (1/6, 16.7%), PR (10/18, 55.6%), and SD (3/11, 27.3%) (*p* = 0.02) (Fig. 4).

### Correlation between the expression level of ROS1, ALK, PD-1, and PD-L1 proteins and the ROS1 gene rearrangement

**Fig. 5:**
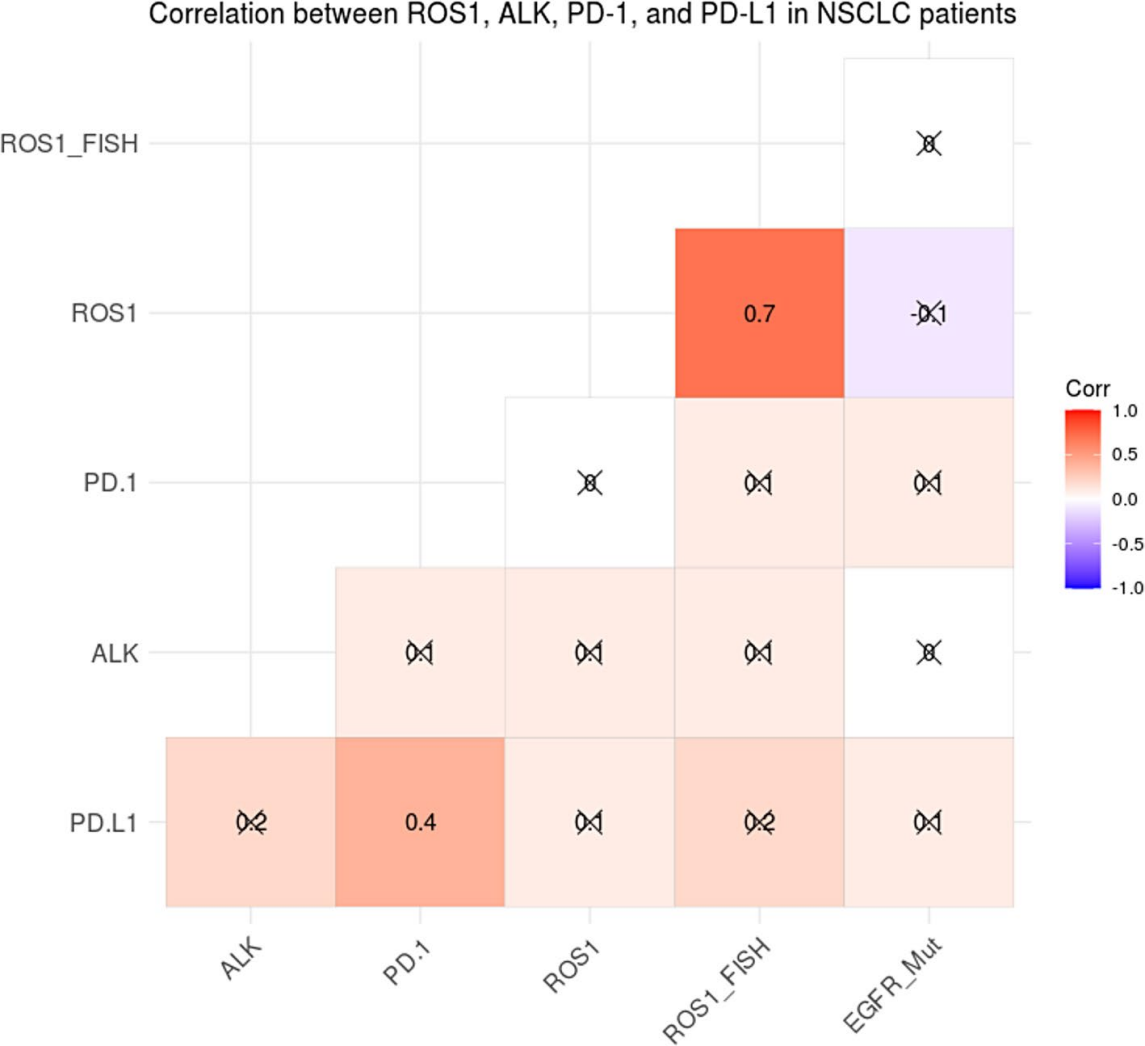
Correlation matrix showing spearman correlations between ROS1, ALK, and PD-1/PD-L1 proteins expression with each other and with *ROS1* gene rearrangement

There was a strong, significantly positive correlation between ROS1 protein expression and *ROS1* gene rearrangement (rho = 0.702, *p* < 0.001). Furthermore, a moderately positive correlation was found between the expression levels of the PD-1 and PD-L1 proteins (rho = 0.420, *p* < 0.001) (Fig. 5).

## Discussion

Lung carcinoma is a leading cause of cancer related death worldwide. Despite the growing body of evidence in research, the molecular mechanisms of lung cancer chemoresistance remains elusive [4]. Thus, accurate biomarker assessment is critical for individually tailored disease management. In the current study, we assessed the clinical prognostic and predictive values of ROS1, ALK*,* PD-1, and PD-L1 protein expressions in NSCLC using IHC along with *ROS1* gene rearrangement using FISH. Our major finding showed that positive ALK expression was significantly associated with poor treatment response and shorter PFS in NSCLC patients. Moreover, both the EGFR mutation and ALK expression were independent predictors for NSCLC.

Since IHC staining for protein expression has the advantages of being less costly and higher in accessibility [18], we examined the of ROS1, ALK, PD1 and PD-L1 proteins expression using IHC in relation to clinic-pathological features of the patients, *EGFR* mutations, survival rates, and response to treatment in a cohort of Egyptian NSCLC patients who were given standard platinum-doublet chemotherapy as a front-line treatment.

Our study revealed that ROS1, ALK, PD-1, and PD-L1 protein expression levels were 18.57%, 54.29%, 84.29%, and 87.14%, respectively, in NSCLC patients. In 15.71% of patients, a *ROS1* gene rearrangement was found. The current study showed a high level of PD-1/PD-L1 expression along with a low level of ROS1 expression. This is consistent with previously published data. In Mahoney and Atkins’ study, the prevalence of PD-L1 protein expression in NSCLC ranged from 24 to 60% [19].

The increased expression of PD-1 and PD-L1 in our current cohort of NSCLC patients compared to previous studies may be explained by the fact that 100% of our study cohort had AC, 61.4% had advanced disease stage (III–IV), and 72.9% were male. Since a previous study by Sorensen, Zhou [20], demonstrated that PD-L1 expression was present in 75% of patients with advanced NSCLC and that higher levels of PD-1 expression were observed in male smokers with adenocarcinoma [21], this could confirm the higher levels of PD-1/PD-L1 expression observed in our study.

In the present study, no significant association was found between PD-1/PD-L1 expression and any of the studied clinicopathological characteristics which was consistent with the previous study Furthermore, they have discovered non-significant relationship between PD-L1 expression and relevant clinicopathological features of the patients as described in our study. Other studies have found that PD-L1 is highly expressed in male smokers with high histologic grade, positive lymph node metastasis, and advanced stage [18, 22].

Till now, the prognostic significance of PD-L1 is still obscure. PD-L1 expression has been associated with a favorable prognosis, a poor prognosis, or no prognostic significance in a variety of studies [23]. The current use of non-standardized IHC techniques to determine the levels of PD-L1 protein in tissue may explain some of these discrepancies. Additionally, PD-L1 expression may vary between lung carcinoma cohorts due to the presence of histological subtypes and different patient selection criteria [24].

We found no significant association between PD-1/PD-L1 expression and patient PFS or response to chemotherapy. This could be explained by the fact that all the patients in our study had been diagnosed with adenocarcinoma on histological examination. The adjusted HRs for PD-1 and PD-L1 positive groups were 1.85 (95% CI: 0.81–4.26; median PFS, 15 months, *p* = 0.24) and 1.11 (95% CI: 0.40–3.08; median PFS, 17 months, *p* = 0.31), respectively, when compared to PD-1/PD-L1 negative groups. Additionally, a previous study found a stronger correlation between PD-L1 expression and survival outcome in patients with squamous cell carcinoma compared to adenocarcinoma [20]. Consistent with the current findings, a previous meta-analysis study involving 64 patients found no statistically significant relationship between PD-L1 expression and survival outcome [25]. On the contrary, other meta-analyses have discovered a strong association between PD-L1 overexpression and poor survival outcomes [26].

A complex relationship may exist between oncogenic driver mutations and PD-1/PD-L1 expression. It has been reported that positive PD-L1 expression was not associated with major oncogenic driver mutations like *EGFR*, *ALK*, *KRAS*, and *BRAF* in NSCLC patients [27]. However, a study by D’Incecco, Andreozzi [21], has linked PD-1/PD-L1 expression to *EGFR* and *KRAS* mutations. In the current study, there was no relationship between PD-1 or PD-L1 expression and *EGFR* mutation, ROS1, or ALK positive expression. Differences in the used staining antibodies, scoring criteria, oncogene analyzed, and mutation rates among patients of different ethnicities may explain these contradictory results. Due to the small number of mutation positive cases, more research is required to confirm our preliminary findings.

The oncogenic ROS1 protein is overexpressed on the tumor cells in most malignant tumors tougher with *ROS1* fusions. *ROS1* rearrangement has been identified as a druggable target in the NSCLC patients, among others [28]. However, the prognostic value of ROS1 protein in NSCLC and its potential therapeutic significance have received relatively little attention until now. The frequency of ROS1 protein expression and *ROS1* rearrangement in the current study were 18.57% and 15.71%, respectively, which is higher in comparison to other previous studies. *ROS1* rearrangements were found in 0.9–1.7% of NSCLC patients in the study by Chen, Hsieh [29]. This could be partly explained by the fact that *ROS1* fusions are common in adenocarcinoma [30], since all of the patients in this study had NSCLC adenocarcinoma.

*ROS1* kinase overexpression has also been discovered in primary and recurrent NSCLC, and it has a potential role as an independent prognostic factor for survival in adenocarcinoma NSCLC cases [31]. In contrast, we found no significant association between *ROS1* protein expression and survival outcomes of NSCLC patients. Most studies in literatures have shown that *ROS1* fusions are mutually exclusive in relation to *EGFR*, *KRAS* mutations, or *ALK* fusions [32, 33]. In concordance with these studies, using spearman correlation, we found no significant correlation between ROS1 protein expression and *EGFR* mutations or ALK protein expression suggesting that ROS1-positive NSCLC patients do not benefit from *EGFR* tyrosine kinase inhibitor therapy. Furthermore, we found no significant relationship between ROS1 expression and gender, smoking status, or tumor stage. The data in this context contrasts to previous findings which show that *ROS1* rearrangement was common in female patients, nonsmokers, and patients with advanced disease stage and triple wild-type *EGFR/KRAS/ALK* genotype [32]. Based on this data, the clinicopathological features of NSCLC patients with positive ROS1 expression differ across ethnicities [34].

Thus, patient choice, which strongly depends on the clinicopathological features, would be ineffective for detecting *ROS1* genetic alterations because the incidence of *ROS1* alterations in NSCLCs is extremely low. Therefore, the molecular tests cannot be used on all patients and an effective screening method is highly required. Since *ROS1* rearrangement occurs at a rate of 0.5 to 2% in NSCLC, IHC appears to be a cost-effective screening assay, allowing for rapid results at a lower cost. Since FISH using dual color “break-apart” probes is the “gold standard” for detecting *ROS1* gene rearrangement [35], we used spearman correlation to examine the correlation between FISH and IHC approaches for detecting *ROS1* rearrangement. We discovered a strong positive correlation between ROS1 protein expression and *ROS1* gene rearrangement (rho = 0.702, *p* < 0.001). In consistent with our findings, a study, conducted by Shan, Lian [35], compared IHC to FISH and real-time RT-PCR in 60 lung adenocarcinomas, including 16 with ROS1 protein expression. Their results indicated that 75–100% of patients with tumors having positive IHC scores were also FISH positive. Consequently, IHC could be considered as an effective and convenient technique for detection of *ROS1* rearrangement in NSCLC rather than the expensive, more difficult, time-consuming, and high expertise demanding molecular tests. However, further studies are needed to compare between the sensitivity and specificity of the two techniques.

Despite the fact that patients with *EGFR* mutations and *ALK* rearrangements shared several clinicopathological features, such as never or light smoking status and adenocarcinoma histology, a previous meta-analysis revealed that *ALK* gene rearrangement was mutually exclusive for *EGFR* mutations, implying a distinct genetic subtype of lung adenocarcinoma [36]. In line with this finding, we found no significant correlation between *EGFR* mutations and positive ALK expression, implying that ALK expression is mutually exclusive of *EGFR.* Furthermore, we did not find any relation between ALK expression and gender or smoking status. Since all the NSCLC patients assessed here were adenocarcinoma, we were unable to detect the relationship between ALK expression and histological subtype. However, we found that positive ALK expression was significantly associated with advanced T stage and left-sided tumors.

We also found that *EGFR* mutations and positive ALK expression were significantly associated with shorter PFS in NSCLC patients. Furthermore, positive LN metastasis, *EGFR* mutations, and ALK expression were independent predictors for PFS in NSCLC patients in a multivariate survival analysis. A multi-variate analysis study by Ito, Miyata [37], demonstrated poor survival outcome and increased rate of metastatic recurrence in *EGFR*-mutated NSCLC patients which is consistent with our findings.

The findings of our study should open the path for future prospective studies of the predictive and prognostic role of PD1/PD-L1 expression along with ROS1 and ALK as measured by immunohistochemistry in patients with NSCLC treated with chemotherapy. Such studies could yield interesting results, particularly regarding the efficacy of immunotherapy and/or receptor tyrosine kinase (RTK) inhibitors in combination with chemotherapy. Because the immune system is so dynamic, future studies should include repeat biopsies during treatment and at progression, as well as sequential analysis of circulating biomarkers.

This study was limited in that it included only adenocarcinoma NSCLC patients, the most common histopathological subtype among Egyptian population. Our study will also open the way for future studies using molecular technique (RNA and/or DNA) to validate the IHC results.

## Conclusions

This study suggests that ALK expression and *EGFR* mutations are independent predictors of NSCLC, emphasizing the rapidly increasing necessity of precision medicine in the treatment of NSCLC. Positive ALK expression was significantly associated with advanced stage and left sided tumors. Despite PD-L1 expression is a strong prognostic marker in patients with NSCLC, the prevalence of PD1/PD-L1 expression found in this study suggests that a significant proportion of patients with NSCLC have positive PD-L1 expression, making them potentially responsive to immunotherapy. There was no significant association between PD-1/PD-L1 and ROS1 expression and the clinicopathological features of NSCLC patients. Further large-scale studies are needed to evaluate PD-L1 expression in relation to other immune check points as well as oncogenic driver mutations among different histopathological subtypes of NSCLC. Furthermore, more research into alternative approaches and assessment criteria for the detection of ROS1 expression and rearrangement is required before it can be used in clinical practice.

## Data Availability

Data available upon request.

## Abbreviations

ALK: Anaplastic lymphoma kinase
CR: Complete response
CT: Computed tomography
CTCAE: Common Terminology Criteria for Adverse Events
DAB: Diaminobenzidine
DAPI: 4′,6-Diamidino-2-phenylindole
EGFR: Epidermal growth factor receptor
FFPE: Formalin-fixed paraffin embedded
FISH: Fluorescence in situ hybridization
IHC: Immunohistochemistry
IRB: Institutional Review Board
LN: Lymph node
NCI: National Cancer Institute
NSCLC: Non-small cell lung carcinoma
OS: Overall survival
PD: Progressive disease
PD-1: Programmed death protein-1
PD-L1: Programmed death ligand-1 (PD-L1)
PFS: Progression-free survival
PR: Partial response
RECIST: Response Evaluation Criteria in Solid Tumors
ROS1: c-ros oncogene1 (ROS1)
RTK: Receptor tyrosine kinase
SD: Stable disease
SSC: Saline sodium citrate
